# Single‐port laparoscopic‐assisted ovariohysterectomy with a modified glove‐port technique in dogs

**DOI:** 10.1111/vsu.13242

**Published:** 2019-06-03

**Authors:** Nina D. Bydzovsky, Barbara Bockstahler, Gilles Dupré

**Affiliations:** ^1^ Department for Small Animals and Horses, Small Animal Surgery University of Veterinary Medicine Vienna Vienna Austria

## Abstract

**Objective:**

To describe a single‐port laparoscopic‐assisted ovariohysterectomy (LOHE) with a modified glove‐port technique in dogs and compare it with previously published laparoscopic techniques for LOHE in dogs.

**Study design:**

Prospective clinical study and technique description.

**Animals:**

Forty‐two healthy female dogs.

**Methods:**

Laparoscopic‐assisted ovariohysterectomy was performed with a custom‐made single‐port device. The total duration of surgery from first incision to skin closure was compared with previously published durations of LOHE in dogs. Short‐term complications were recorded.

**Results:**

The median total duration of surgery was 24 minutes (range, 17.5–39.5; mean, 25.73; SD, 6.12), which was shorter than that described in most previously reported studies of LOHE in dogs (range, 20.8 ± 4.00–60.0 ± 18.45 minutes; *P* < .001). Intraoperative complications were minor, but wound complications occurred in 12 of 42 (29%) dogs.

**Conclusion:**

Single‐port LOHE with the glove‐port technique in combination with a wound retractor and nonarticulated instruments was completed in all dogs. This technique was faster than what has been previously reported for other LOHE, but local wound complications were common.

**Clinical relevance:**

The glove‐port technique described here offers a low‐cost alternative to other commercially available single‐port devices.

## 1 INTRODUCTION

Laparoscopic surgery has gained popularity in human and veterinary surgery because of its reduced postoperative pain, faster recovery with shorter hospital stay, and superior cosmetic outcome.^1^, ^2^, ^3^, ^4^, ^5^ Laparoscopic sterilization has become a widely available alternative to open laparotomy^1^, ^2^, ^3^, ^4^, ^5^, ^6^, ^7^, ^8^, ^9^, ^10^, ^11^, ^12^, ^13^, ^14^, ^15^, ^16^, ^17^, ^18^, ^19^, ^20^, ^21^, ^22^, ^23^, ^24^, ^25^, ^26^, ^27^, ^28^, ^29^ in dogs and can be achieved via laparoscopic ovariectomy (LOE)* or laparoscopic ovariohysterectomy (LOHE).^†^ Reducing the number of ports in laparoscopic surgery minimizes tissue trauma and improves recovery in human^30^, ^31^, ^32^, ^33^, ^34^, ^35^, ^36^ and veterinary^1^, ^2^, ^5^, ^12^, ^14^, ^16^, ^24^, ^37^ medicine. Single‐port systems, such as single‐incision laparoscopic surgery (SILS), R‐Port (Advanced Surgical Concepts, Wicklow, Ireland), TriPort (Advanced Surgical Concepts), or GelPort (Applied Medical Systems, Rancho Santa Margarita, California), allow simultaneous entry and manipulation of the scope, and several instruments have been developed in human medicine for various indications.^32^, ^33^, ^38^, ^39^ Single‐port LOE techniques have consequently been developed in veterinary surgery.^‡^ Most published techniques for LOHE involve the use of 3 or 4 ports.^§^ Two‐port techniques^2^, ^20^ and transvaginal approaches^8^, ^10^, ^23^ have recently been described, commercially available single‐port multiaccess devices for LOHE have been used in only 2 studies.^5^, ^27^

The glove‐port technique was first described by Khiangte et al^34^ as an alternative to a single‐port device. This approach was designed for cost efficiency and has been applied

^*^References 1, 4, 12, 13, 14, 16, 18, 19, 21, 22, 24, 25, 26.

*^†^References* 2, 3, 5, 6, 7, 8, 9, 10, 11, 13, 15, 17, 20, 23, 27, 28, 29.

*^‡^References* 1, 12, 14, 16, 19, 21, 22, 24.

*^§^References* 3, 6, 7, 8, 11, 13, 15, 17, 28, 29.

in many laparoscopic procedures in man.^30^, ^31^, ^34^, ^35^, ^36^, ^40^, ^41^, ^42^, ^43^, ^44^ Our team successfully treated canine pyometra with a laparoscopic‐assisted modified glove‐port technique and nonarticulated instruments.^9^ However, we are not aware of any reports describing use of the glove‐port technique as an alternative to commercially built single‐port systems for elective LOHE in dogs.

Therefore, the present study evaluated the feasibility of LOHE with the glove port and nonarticulated instruments and compared the surgical duration and short‐term complication rates of LOHE with a glove‐port technique and nonarticulated instruments to those of previously published laparoscopic techniques. We hypothesized that conversion from LOHE to open laparotomy by using this technique would be minimal (<5%) and comparable to that in previous studies and that the total duration of the procedure would be not exceed that described in reports of previous studies.

## 2 MATERIALS AND METHODS

This study was approved by the University of Veterinary Medicine Vienna institutional ethics committee in accordance with Good Scientific Practice guidelines and Austrian national legislation (protocol No. 03/02/97/2014). Dogs were enrolled in the study with owners’ consent.

### 2.1 Dogs

Dogs with a minimum age of 6 months and a minimum body weight of 5 kg were included. Each dog underwent thorough clinical and gynecological examination including vaginoscopy, vaginal smear, and ultrasonographic examination. Exclusion criteria included any contraindications for laparoscopic surgery as well as intra‐abdominal or gynecological abnormalities discovered during clinical and ultrasonographic examination. Preoperative blood work included a minimum database of hematocrit, total solids, and creatinine. Complete blood count and albumin were obtained in most cases (34/42). Age, breed, weight, body condition score, previous surgeries, and ASA class were recorded.

### 2.2 Anesthesia

Dogs were randomly assigned to premedications and constant‐rate infusion protocols as part of another study. Premedication included (1) methadone (Methadon Streuli 10 mg/mL; Streuli Pharma AG, Uznach, Switzerland; 0.1–0.2 mg/kg IV or IM) and acepromazine (Vanastress 10 mg/mL injection solution; Vana GmbH Vienna, Austria; 0.01–0.02 mg/kg IV or IM); (2) remifentanil (Ultiva 1 mg/mL injection solution; GlaxoSmithKline Pharma GmbH, Vienna, Austria; 0.06 mg/kg IV or IM) and acepromazine (0.01–0.02 mg/kg IV or IM); or (3) sufentanil (Sufenta injection solution 50 μg/mL, Janssen‐Cilag Pharma GmbH, Vienna, Austria; 0.006 mg/kg IV) and acepromazine (0.01–0.02 mg/kg IV).

Anesthesia was induced with propofol (propofol “Fresenius” 1%; Fresenius Kabi GmbH, Graz, Austria; 4 mg/kg IV) and maintained with isoflurane in oxygen. A constant‐rate infusion of fentanyl (50 μg/mL; Janssen‐Cilag Pharma GmbH; 0.01–0.02 mg/kg/h), remifentanil (0.01 mg/kg/h), or sufentanil (0.02 mg/kg/h) was initiated for analgesia at the beginning of maintenance and continued until the end of surgery. All dogs were ventilated with a volume‐controlled ventilation mode with a tidal volume of 12 mL/kg, maximal pressure of 15 cm of H_2_O, and a frequency of 14 breaths/minute. Monitoring included capnography, electrocardiography (ECG), body core temperature, pulse oximetry, and noninvasive blood pressure.

### 2.3 Assembly of the glove port

**Figure 1:**
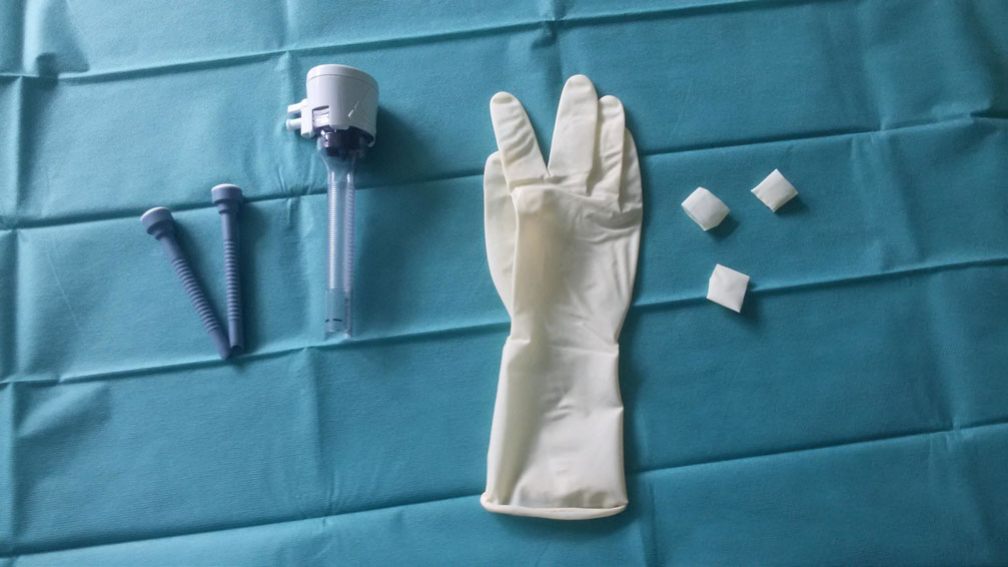
Components of the glove‐port

The modified glove‐port technique was prepared as described by Khiangte et al.^34^ A pair of size 6 1/2 surgical gloves (Vasco OP Sensitive; B. Braun Melsungen AG, Melsungen, Germany), two 5‐mm laparoscopic cannulas (Apple Hunt; Apple Medical Cooperation, ASF‐Medical GMBH, Teesdorf, Austria), one 10‐mm cannula (Kii Optical Access System; Applied Medical, Salzburg, Austria), and a small Alexis wound retractor (for incisions from 2.5 to 6 cm; Applied Medical) were used (Figure 1).

**Figure 2:**
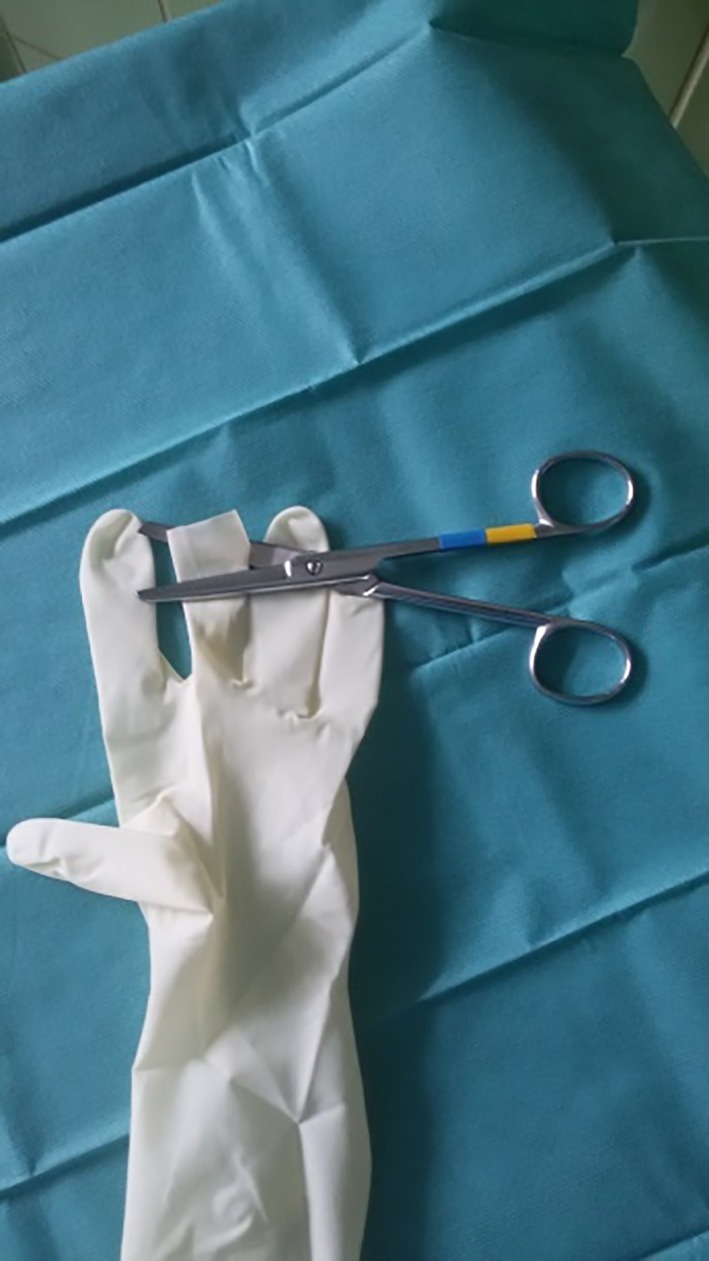
One finger was cut into strips

**Figure 3:**
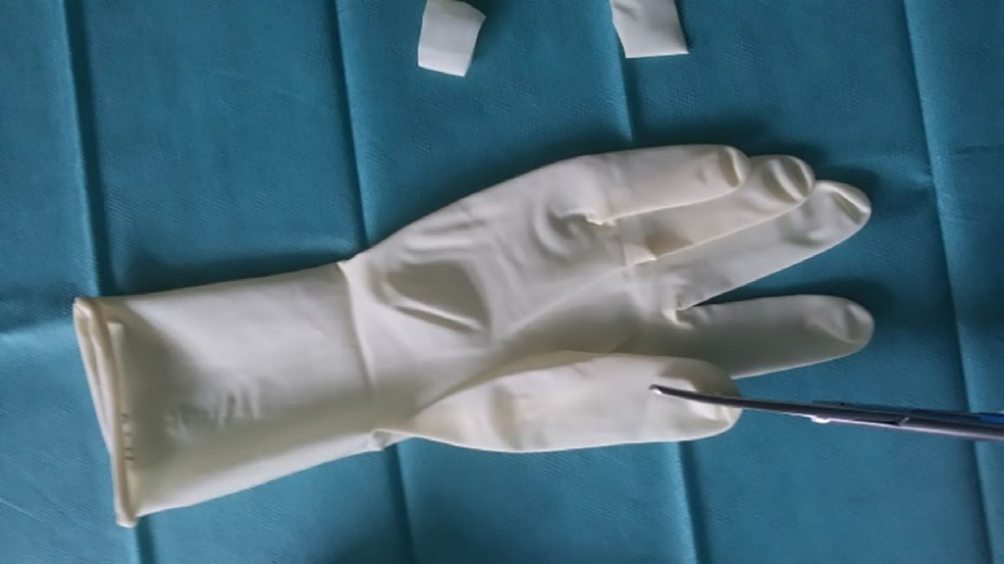
Small longitudinal incisions were made at the fingertips of the third and fifth fingers

**Figure 4:**
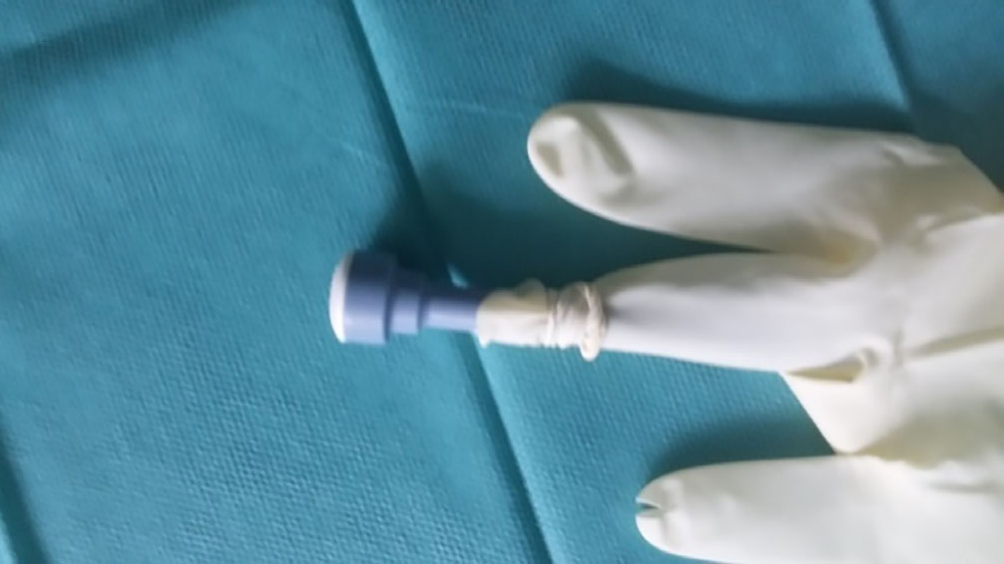
Five‐millimeter cannulas were inserted and fixed with rubber bands made from the other surgical glove

**Figure 5:**
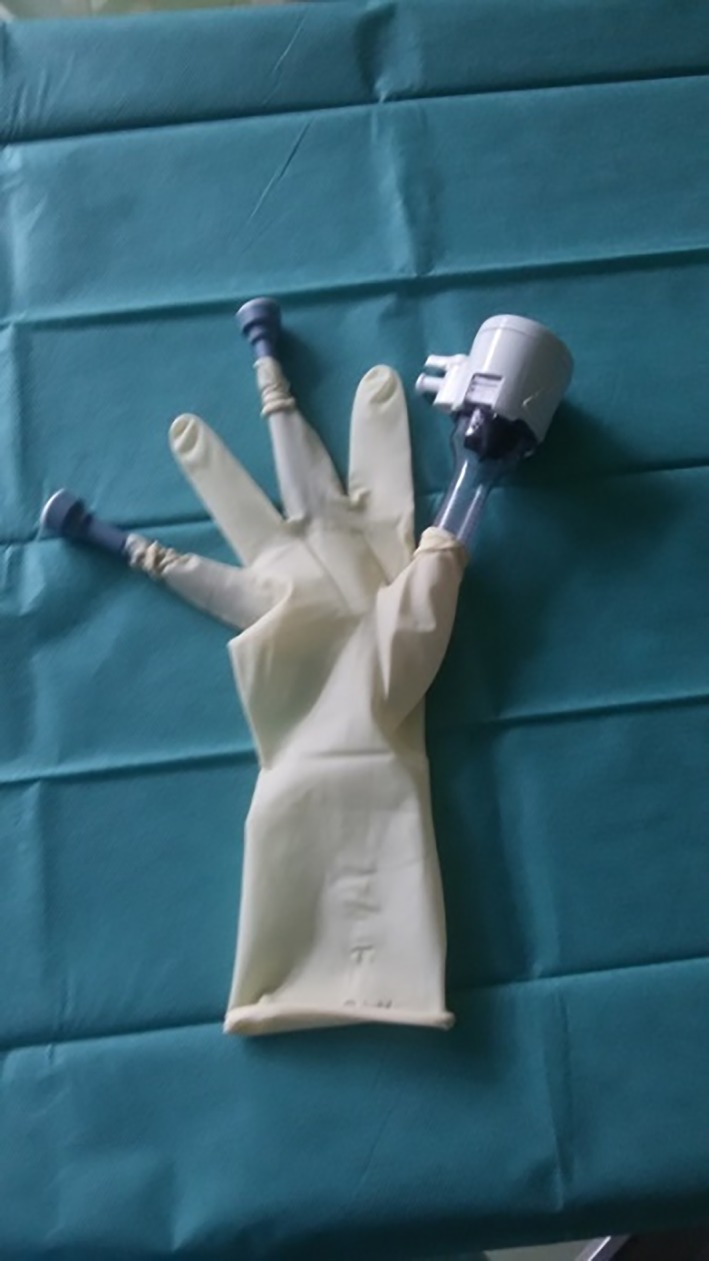
A 10‐mm cannula was inserted into the thumb of the surgical glove and fixed with a rubber band

One finger of the surgical glove was cut into strips approximately 1 cm in width for use as rubber bands (Figure 2). Two small longitudinal incisions were made at the fingertips of the little and middle fingers of another surgical glove, and the 5‐mm cannulas were inserted and fixed with the previously created rubber bands (Figures 3, 4). A 10‐mm cannula was inserted into the thumb of the surgical glove and fixed with a premade rubber band to enable the passage of a 10‐mm vessel‐sealer‐divider device (Figure 5).

### 2.4 Surgery

The same surgeon and assistant performed all surgeries in the surgical department of the Veterinary Medicine University of Vienna. Although the first surgeon (GD) was an experienced laparoscopic surgeon, none of the surgeons had experience with this technique before.

The dog was placed in dorsal recumbency on a tiltable table (TT endoscopic positioner; Apex Veterinary Equipment, Englewood, Colorado). The bladder was emptied manually, and the ventral abdomen was aseptically prepared. The monitor was located at the caudal end of the table throughout the surgery. The distance between the pubic brim and umbilicus was measured with a ruler, and a 2.5–3‐cm skin incision was made on the ventral midline at the junction of the middle and caudal third of this distance. The linea alba was punctured, and a minilaparotomy was performed. The flexible ring of the Alexis wound‐retractor was inserted through the incision into the abdominal cavity.

**Figure 6:**
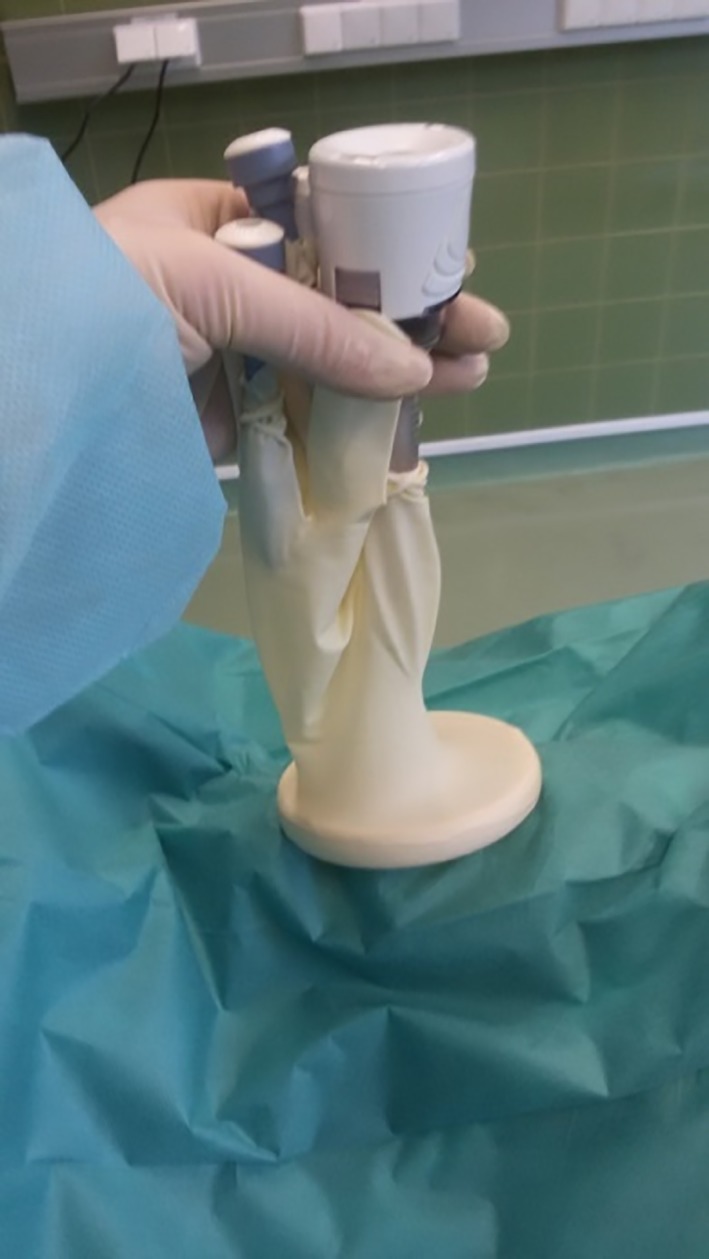
The glove prepared with 3 cannulas was secured over the outer ring

**Figure 7:**
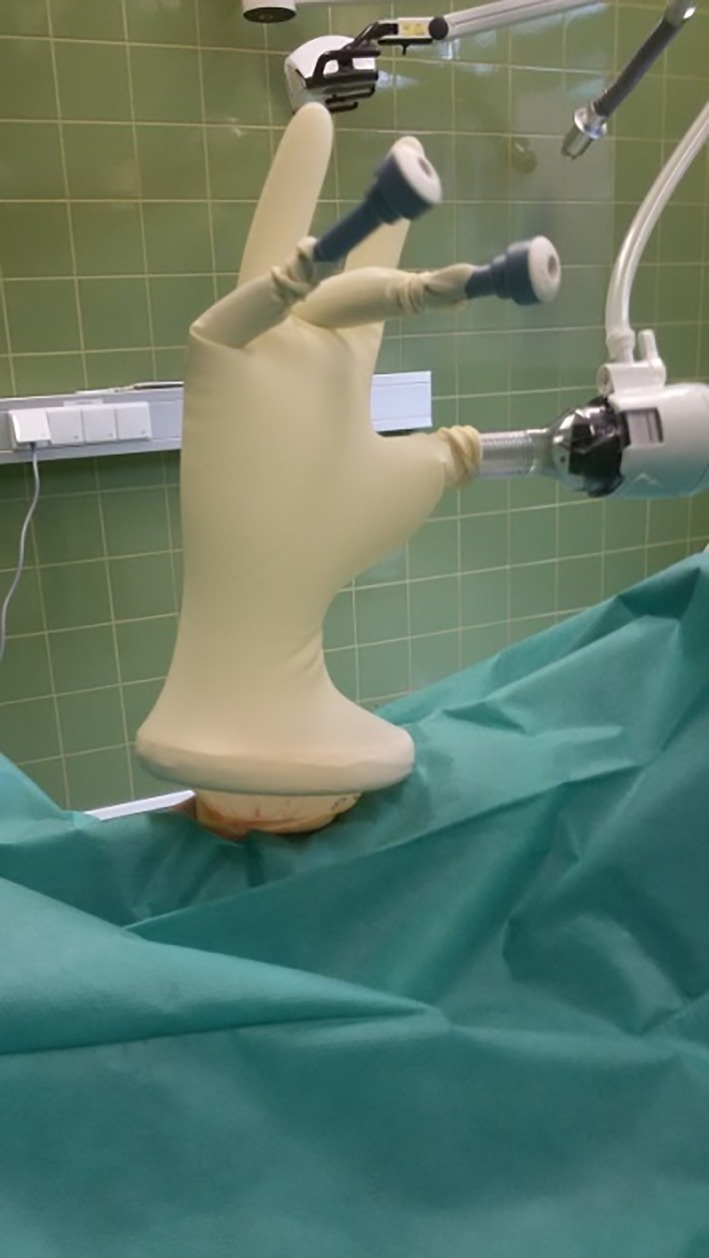
Pneumoperitoneum with a pressure of 10 mm Hg was established via insufflation of CO_2_

The glove that had been prepared with 3 cannulas was secured over the outer ring with the thumb cranial, and tight adherence was ensured. Pneumoperitoneum with a pressure of 8–10 mm Hg was established through the 10‐mm cannula via insufflation of CO_2_ (Electronic CO_2_‐ Endoflator; Karl Storz GMBH KG, Tuttlingen, Germany; Figures 6, 7).

**Figure 8:**
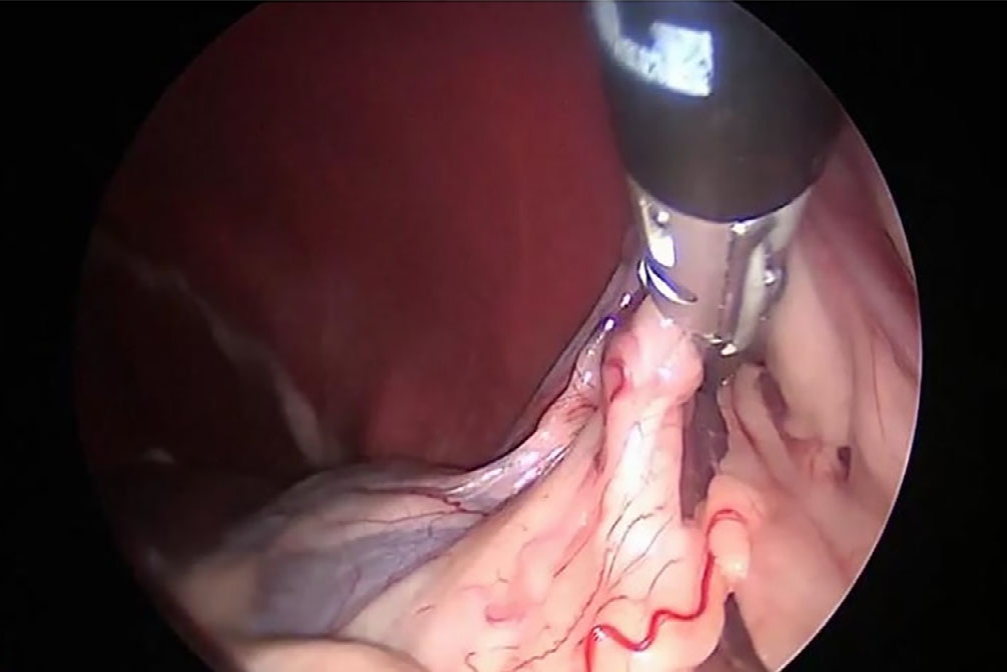
Sealing and division of the suspensory ligament and the broad ligament of the left ovary

A 5‐mm 30° laparoscope (Hopkins II; Karl Storz) was inserted through the caudal cannula (little finger of the surgical glove), and the abdominal cavity was inspected thoroughly. Grasping forceps (MANHES grasping forceps; Karl Storz) were inserted through the cannula in the middle finger of the surgical glove, and a 10‐mm LigaSure Atlas (LigaSure V; Valleylab, Covidien, Vienna, Austria) was inserted through the 10‐mm cannula in the thumb of the glove. Surgeons were on the right side of the dog, and the surgical table was rotated 45° to the right to facilitate visualization of the left ovary. The suspensory ligament was grasped and lifted with grasping forceps, and the broad ligament was sealed and divided by using the LigaSure device (Figure 8).

**Figure 9:**
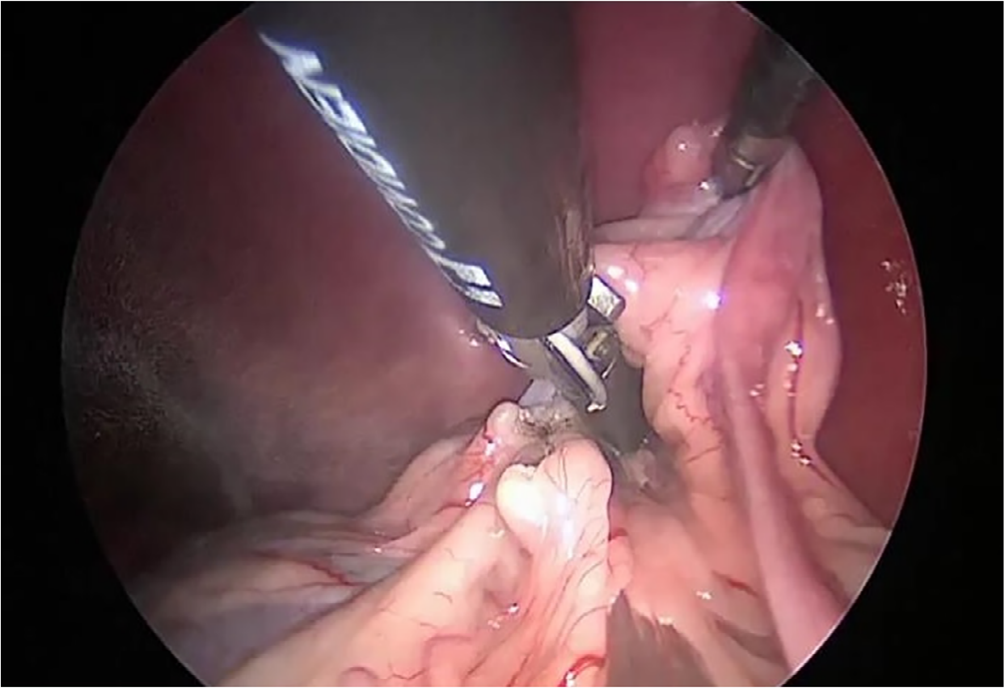
Sealing and division of the suspensory ligament and the broad ligament of the right ovary

**Figure 10:**
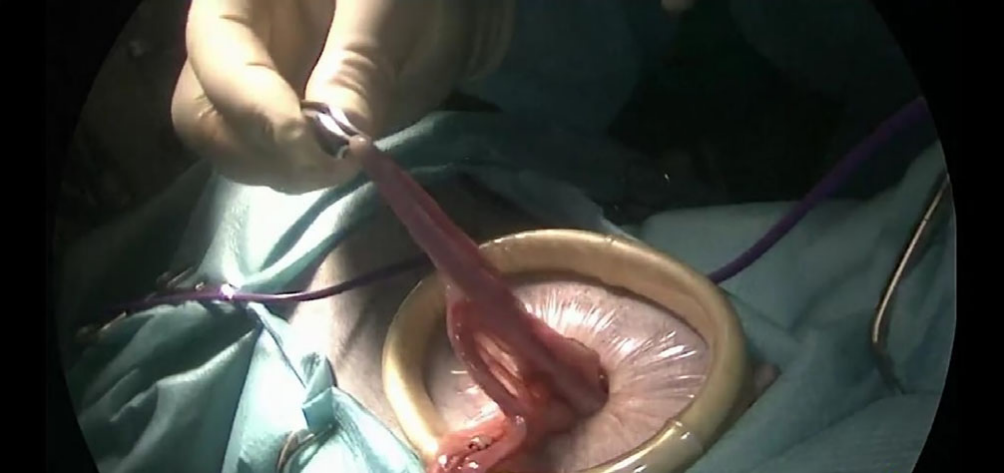
Removal of the uterus from the abdomen with the Alexis wound retractor

The table positioner was titled 45° toward the left side, and the same procedures were repeated for the right ovary (Figure 9). The patient was returned to dorsal recumbency while the grasping forceps held the right ovary. The surgical glove was disconnected from the Alexis, and the ovaries and the uterus were pulled out of the abdomen through the wound retractor (Figure 10).

**Figure 11:**
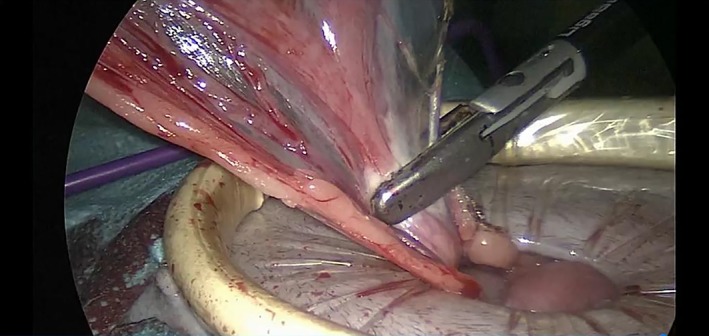
Transection of the cervix with the LigaSure

The cervix diameter was measured, and the uterus was divided at the cervix by using the LigaSure device (Figure 11). The Alexis wound retractor was removed after completion of the ovariohysterectomy. The abdominal fascia was closed with simple interrupted sutures of 0 to −2/0 monofilament absorbable material (Biosyn; Covidien), the subcutis was gently reapposed and not sutured, and the skin was sutured with −3–0 monofilament nonabsorbable suture material with simple interrupted pattern (Dermalon; Covidien).

### 2.5 Postoperative management

Pain management included a single dose of buprenorphine (Temgesic 0.3 mg/mL; Indivior EU, Berkshire, United Kingdom; 0.01–0.02 mg/kg IV) and meloxicam (Metacam 5 mg/mL; Boehringer Ingelheim, Ingelheim/Rhein, Germany; 0.2 mg/kg IV). Meloxicam (0.1 mg/kg, once daily orally) was continued for 4 days after surgery.

Dogs were sent home with an E‐collar, and suture removal and final examinations were performed 10–14 days postoperatively. Any additional examinations were recorded.

### 2.6 Recorded data

Time to assemble the glove port, beginning with all instruments on the table and the same assistant performing the assembly, was measured in minutes. The interval between the skin incision and insertion of the Alexis wound retractor and the time required for installation of the glove port and establishment of pneumoperitoneum (ie, until the pressure reached 10 mmHg) were recorded in minutes. Time elapsed between grasping the left/right ovary and mobilization of the ovary and time for removal of the glove port, retrieval of ovaries and uterus, and cutting of the cervix were measured in minutes; the same applied to time for extraction of the Alexis wound retractor. Surgical duration from glove‐port insertion until port removal, total duration of surgery from skin incision to closure, duration of anesthesia, and time for wound closure were recorded in minutes. The length of the incision and the cervix diameter were measured in centimeters and, when present, abnormalities of ovaries, uterus, or other abdominal organs were recorded.

Interference between instruments inside the abdomen (“sword fighting”) and interference between operators outside the abdomen were also recorded. Hemorrhage, trauma to intra‐abdominal organs, or conversion were defined as major intraoperative complications; minor bleeding, CO_2_ leakage, interference between operators and sword fighting inside the abdomen were reported as minor complications.

Anesthetic complications included bradycardia (decrease of heart frequency of more than 50% from preanesthetic heart rate), hypotension (mean blood pressure less than 60 mm Hg), hypothermia, hypocapnia, myoclonies, and changes in ECG. Postoperative complications (ie, hematoma, seroma, hernia, swelling, inflammation, and dehiscence of a suture within the first 14 days after surgery) were also recorded.

### 2.7 Literature review

A search for other studies was performed in PubMed, Ovid, Scopus, and ISI WoS from February 2012 until June 2018 with the key words “laparoscopic,” “laparoscopic‐assisted elective OHE,” and “dogs.”

### 2.8 Statistical analysis

All analyses were performed in IBM SPSS v24 (IBM, Armonk, New York). The appropriate aggregated data were weighted by the number of individuals in each study. Data are shown as mean and SD or median and range as appropriate.

The mean total operation time of the other studies was compared to the mean total operation time of our study with 1‐sample *t* test. The assumption of normal distribution was assessed with the Kolmogorov–Smirnov test. *P* < .05 (5%) was considered significant.

## 3 RESULTS

### 3.1 Dogs

Forty‐two female dogs were enrolled, including 3 golden retrievers, 3 Labrador retrievers, 3 border collies, 2 Australian shepherds, 1 each from 11 various breeds, and 20 mixed‐breed dogs. Median body weight was 19 kg, median age was 17 months, and median body condition score was 3 (Table 1).

**Table 1:** Data retrieved from the veterinary literature on LOHE

Authors	Aim of study	Ports, n	Dogs, n	BW, mean, kg	BW, SD, kg	BW, median, kg	BW range from, kg	BW range to, kg	Age, mean, mo	Age, SD, y	Age, median, mo	Age range from, mo	Age range to, mo
Bydzovsky et al^a^	LOHE	1	42	19.17	7.77	19	5.	39.5	23.95	16.07	17	7	81
Austin et al^7^	LOHE	3	9	…	…	17.7	10	26	…	…	…	5	60
Bakthiari et al^8^	Conventional LOHE vs transvaginal LOHE	3	24 (12 in each group)	…	…	…	14	17	…	…	…	…	…
Brun et al^10^	Hybrid NOTES technique with a transvaginal access and 1 port	1 + 1 transvaginal port	1	…	…	…	…	…	…	…	…	…	…
Davidson et al^11^	LOE vs. LOHE	4	16	…	…	17.9	10	38	…	…	10.5	4	36
Devitt et al^2^	Comparison of duration, complications, stress, pain of open OHE vs LOHE	2	10	22.1	5	…	…	…	17.4	9.36	…	…	…
Dutta et al^13^	LOE vs 2 techniques of LOHE (endoclip & electrocautery vs electrocautery)	3	40 (20 in each group)	17.2	0.62	…	15	20	20.68	1.28	…	16	28
Hancock et al^3^	Comparison of postoperative pain after median celiotomy with LOHE	3	8	…	…	11	10.1	12.2	…	…	…	…	…
Kim et al^15^	Evaluation of postoperative pain behavior and biochemical stress response in dogs undergoing LOHE	3	16	19.55	3.65	…	…	…	18.6	1.8	…	…	…
Mayhew and Brown^17^	Comparison of 3 techniques of ovarian pedicle hemostasis during LOHE (sutures vs clips vs vessel sealing)	3	30 (10 in each group)	…	…	20	8.9	35.5	…	…	…	12	48
Pukacz et al^20^	LOHE	2	59	…	…	21.1	3	45	…	…	30	6	138
Sanchez‐Margallo et al^5^	LOHE with SILS port	1	9	11.28	4.63	…	7.5	23	4.55	2.24	…	12	84
Silva et al^23^	Total‐NOTES vs single‐port laparoscopic assisted vs conventional OHE	1 and 1 transvaginal port	40, 12 NOTES, 13 single‐port, 15 conventional	12.6	6.	…	8.9	25	…	…	…	…	…
Wenkel et al^28^	LOHE	3	27	…	…	…	6	48	…	…	…	9	54
Zhang et al^29^	Comparison of IL‐6 and C‐reactive protein in dogs undergoing a gasless LOHE vs conventional LOHE	3 conventional vs 4 gasless	10	…	…	…	10	23	…	…	…	12	24

Abbreviations: …, no data; BW, body weight; IL, interleukin; LOE, laparoscopic ovariectomy; LOHE, laparoscopic‐assisted ovariohysterectomy; NOTES, natural orifice transluminal endoscopic surgery.

a Current study.

### 3.2 Measurements

Time for assembly of the glove port ranged from 0.5 to 3 minutes (median, 1.5; mean, 1.62; SD 0.73; Table 2). Median surgical duration was 14 minutes (range, 9.5–26; mean, 15.89; SD, 4.58), and median total operation time was 24 minutes (range 17.5–39.5; mean, 25.73; SD, 6.12). Median length of the incision was 2.8 cm, median distance between the os pubis and the umbilicus was 19 cm, and the cervix as measured in 31 dogs measured 1 cm in diameter (median).

**Table 2:** Times measured in 42 dogs undergoing LOHE with glove port^a^

Procedure	Mean	SD	Median	Range from	Range to
Assembly of the glove port	1.62	0.73	1.5	0.5	3
Skin incision and insertion of the Alexis wound‐retractor	3.45	1.31	3	0.5	7
Installation of glove port and initiation of pneumoperitoneum until 10 mmHg	1.01	0.34	1	0.5	2
Grasping of the left ovary and mobilization	2.44	1.07	2	1	5
Grasping of the right ovary and mobilization	2.69	1.93	2	1	10
Deinstallation of glove port, retrieval of ovaries and uterus, and cutting the cervix	3.02	1.45	2.75	1	8
Extraction of Alexis wound‐retractor	0.93	0.42	1	0.5	2.5
Closure of surgical wound	5.2	2.4	4	2	14
Total surgical duration from initiation of the port until removal of the port	15.89	4.58	14	9.5	26
Total operation time from skin incision to closure	25.73	6.12	24	17.5	39.5

Abbreviation: LOHE, laparoscopic‐assisted ovariohysterectomy.

a All times are in minutes.

### 3.3 Complications

No major intraoperative complications occurred, nor was there any conversion to laparotomy. Minor bleeding from the ovarian bursa occurred in 4 dogs, and all instances were controlled with the LigaSure device. Interference between the operators outside of the abdomen occurred in 27 cases, and interference of instruments inside the abdomen was noted in 21 cases. These interferences did not affect the overall surgical time (*P* = .632).

Postoperative local wound complications consisting of local inflammation and wound dehiscence occurred in 29% (12/42) of dogs. Placement of sutures was required in 5 dogs (2 of which occurred after 2 and 3 days), whereas all other wounds healed uneventfully after local treatment. Antibiotics (amoxicillin/clavulanic acid, 20 mg/kg twice daily; Kesium, Ceva Tiergesundheit GmbH, Düsseldorf, Germany) were administered in 3 dogs.

### 3.4 Comparison with previous studies

The results of our study were compared to those of 14 previous reports.^2^, ^3^, ^5^, ^7^, ^8^, ^10^, ^11^, ^13^, ^15^, ^17^, ^20^, ^23^, ^28^, ^29^ Mean age (17.4–54.60 months; range 4–138) and median age (10.5 months and 30 months) did not differ from those in the present study (*P* = .869; Table 1). The mean body weight in previous studies (range, 3–48 kg; mean range, 11.28–22.1 kg; and median range, 11 kg‐21.1 kg) did not differ from the body weight in our study (*P* = .859, Table 1). Most previously published studies used more than 1 port (Table 1). Mean total duration of surgery (from first incision to skin closure) in previous studies varied between 20.8 ± 4.00 and 60.0 ± 18.45 minutes, which was longer that than in our study (25.73 ± 6.12 minutes, *P* < .001; Table 3).

**Table 3:** Previous publications reporting duration of surgery^a^

Authors	Surgery	Minimum	Maximum	Operation times, median	Operation times, mean
Austin et al^7^	…	35	100	59.4	60 ± 18.45
Bakthiari et al^8^	LOHE	…	…	…	34.2 ± 4.03
	Transvaginal	…	…	…	37 ± 3.56
Brun et al^10^	…	…	…	94	…
Bydzovsky et al^b^	…	17	39	24	25.72 ± 6.12
Davidson et al^11^	…	47	175	120	…
Devitt et al^2^	…	…	…	…	20.8 ± 4
Dutta et al^13^	Endoclip & electrocautery	…	…	…	50.83 ± 5.3
	Electrocautery	…	…	…	47.17 ± 4.13
Hancock et al^3^	…	…	…	55	…
Kim et al^15^	Control group	…	…	…	36.8 ± 2.4
	Bupivacaine group	…	…	…	37.5 ± 4.4
Mayhew et al^17^	Suture group	62	93	75	…
	Clip group	41	90	53	…
	Vessel sealing group	22	52	36	…
Pukacz et al^20^	…	30	88	59	…
Sanchez‐Margallo et al^5^	SILS LOHE	27	73	52	52.66 ± 15.2
Silva et al^23^	NOTES	…	…	…	25.7 ± 6.8
	Single‐port	…	…	…	23.5 ± 4
Wenkelet al^28^	…	35	125	57	…
Zhang et al^29^	LOHE	45	75	55	…
	Gasless LOHE	50	82	60	…

Abbreviations: …, no data; LOHE, laparoscopic‐assisted ovariohysterectomy; NOTES, natural orifice transluminal endoscopic surgery; SILS, single‐incision laparoscopic surgery.

a All times are in minutes.

b Current study.

Although most studies did not report local wound complications after single‐port or multiport surgeries, our findings are closest to those reported by Gonzales‐Gasch and Monnet,^14^ with a 18% local wound complications rate.

## 4 DISCUSSION

In this study, LOHE with a modified glove‐port technique was completed in all 42 dogs, prompting us to accept our hypothesis that the rate of conversion from laparoscopic to open laparotomy would be minimal (<5%). Median and mean total durations of LOHE with the glove‐port technique and nonarticulated instruments were comparable or shorter than those reported in previous studies (Table 3). We are aware of only 1 study comparing natural orifice transluminal endoscopic surgery (NOTES), conventional OHE, and single‐port laparoscopic‐assisted OHE in which the median surgical time with a SILS port was slightly faster than that in our study.^23^

The slight difference in time required to assemble the glove port (Table 2) for the first 21 dogs compared with for the last 21 dogs is consistent with a learning curve. Port assembly has previously been reported to take 4–8 minutes,^34^, ^36^, ^44^ and some authors suggest that the time to construct the device will prolong operative times compared with use of commercial single‐port devices.^30^, ^41^, ^44^ However, this was not detected in our study.

The mean time from initiation of pneumoperitoneum to full skin closure with the glove‐port technique (19.8 minutes) compares favorably with previous laparoscopy‐assisted studies by Devitt et al^2^ (20.8 ± 4 minutes) and Silva et al^23^ (20.8 ± 6.9 minutes). Reducing the number of ports and the use of single‐access ports have been proposed to reduce surgical duration. Indeed, use of a SILS port rather than a multiport reduced surgical duration for gastropexy, ovariectomy, or both in 98 dogs.^14^ Similar findings were observed when comparing a single‐portal splenectomy with a 3‐portal laparoscopic splenectomy in 18 dogs.^37^ Only a few studies with a limited number of cases have evaluated LOHE in dogs (Table 3). Most of these studies* used 3 or 4 ports. A reduced number of ports with transabdominal suspension sutures and an operative laparoscope with a working channel^2^ or even a transvaginal approach have alternatively been used.^8^, ^10^, ^23^ Wallace et al^27^ reported a laparoscopy‐assisted OHE with a SILS port in 7 dogs with mucometra or pyometra less than 5 cm in diameter. Our team used a modified glove‐port technique and nonarticulated instruments for the treatment of pyometra with a diameter up to 7 cm in dogs,^9^ with a median surgical time of 57 minutes. Silva et al^23^ reported that the mean duration of NOTES and single‐port laparoscopy were shorter than that in a conventional OHE group. Tapia‐Araya et al^24^ did not detect differences in *References 3, 6, 7, 8, 11, 13, 15, 17, 28, 29 surgical duration between procedures with a SILS‐port LOE (n = 5) and those with a 3‐portal LOE (n = 5). The single‐port technique that will ultimately most minimize surgical duration remains unknown.

No major intraoperative complications occurred, and no conversions were required in our study. Intraoperative complication rates in laparoscopic surgeries range from 2% to 35%,^14^, ^19^, ^45^ and a conversion rate of 23% has been reported.^45^ In the study in which multiport was compared with SILS,^14^ all intraoperative complications (n = 12/98) and all required conversions (n = 3/98) were observed in the multiport group. Surgical complications in an early study of OHE^11^ included mild hemorrhage from the stump after ligation and transection with bipolar electrocauterization (n = 4/16) and splenic laceration (n = 3/16). Splenic laceration was also common in other studies^3^, ^17^ and increased the duration of surgery because bleeding impaired visualization. Four cases of minor bleeding from skin incision, omentum, or ovaries were observed in our study; these complications were all controlled with the LigaSure. The open approach used for the glove‐port technique dramatically limits the risks of iatrogenic damage associated with trocar entries. The glove was always positioned 2/3 of the distance between the umbilicus and the pubic brim, which enabled sealing of the suspensory and broad ligaments and exteriorization of the uterus through the same hole.

We used straight instruments working parallel to the scope, which resulted in operator interferences and sword fighting, as reported previously.* However, only one 10‐mm instrument was used (LigaSure), and intra‐abdominal interference of instruments never impeded surgery. The flexibility of the glove port allows high maneuverability of *References 5, 10, 13, 17, 24, 25, 28, 32, 34, 35, 36, 38, 42, 44.

the instruments compared to the narrow cylinder in commercial single‐port systems. The alternative use of a glove‐port device without cannulas^31^ or articulating instruments may additionally reduce operator interference and sword fighting.* A 10‐mm LigaSure device was used to seal vessels and section tissues of the suspensory ligament, mesovarium, broad ligament and cervix, shortening operative times.^1^ Methods to achieve hemostasis and sectioning of the suspensory and broad ligaments include use of monopolar^13^, ^25^ and bipolar electrocoagulation,^†^ laser,^26^ endoclips^10^, ^13^, ^17^ ultrasonic scalpel,^3^, ^7^ LigaSure, and other vessel‐sealing devices.^1^, ^17^, ^18^ These methods generally shorten surgery times. The LigaSure device was successfully used to coagulate the uterine arteries and seal cervix up to 1.8 cm in diameter. Sanchez‐Margallo et al^5^ also sealed and sectioned the cervix with LigaSure in 9 dogs without any complications. One in vitro study investigated a bipolar vessel‐sealing device (LigaSure) for the sealing of uterine bodies up to 9 mm in diameter.^46^ The long‐term consequences of sealing versus ligation of the uterine body are not known. Austin et al^7^ used a harmonic ultrasonic scalpel that allowed a maximal vessel size to safely transect only 2 mm. However, ligatures were needed, which may have contributed to longer surgical times.

Postoperative complications after LOHE include vaginal discharge, fever, lethargy, anorexia, pseudopregnancy, bleeding from surgical wounds, wound inflammation and partial skin dehiscence.^11^, ^19^, ^20^, ^23^ Twenty‐nine percent of dogs (12/42) in our study presented postoperatively with wound complication, from local inflammation to minor

*References 5, 24, 28, 32, 33, 35, 36, 38, 44.

*^†^References* 2, 8, 11, 20, 23, 25, 26, 29.

*^‡^References* 4, 5, 6, 9, 12, 16, 18, 21, 22, 24, 27, 28.

skin dehiscence. Although most studies do not mention postoperative complications, a local wound infection rate of 18% was found in 1 study.^14^ Wound infections were common when port site incisions were closed with n‐butyl‐cyanoacrylate.^19^ The high frequency of wound complications in our study could reflect poor recommendations by owners or compliance and licking, as previously suggested^11^, ^27^; however, the influence of the pressure of the plastic on the skin margins combined with electrocautery to cut the skin warrants additional investigation.

The single‐port glove‐port technique has previously been used in man and offers several advantages compared with commercially available single‐port systems.^30^, ^31^, ^34^, ^35^, ^36^, ^40^, ^41^, ^42^, ^43^, ^44^ The material used is cost effective,^9^, ^30^, ^31^, ^34^, ^35^, ^36^, ^40^, ^41^, ^42^, ^43^, ^44^ which prompted its proposed application in developing countries.^9^, ^30^, ^34^, ^36^, ^40^, ^43^, ^44^ Although it was designed for single use, the Alexis wound retractor with the trocars can be reused several times after sterilization. Up to 5 instruments can be inserted into the glove and used simultaneously or alternatively.^34^, ^36^, ^40^, ^41^, ^43^, ^44^ The range of motion of the scope and instruments compares favorably with that achieved with commercially available ports.^30^, ^31^, ^34^, ^40^, ^43^, ^44^ Some surgeons attribute this finding to the elasticity of the surgical glove,^36^, ^40^ but several factors, such as the instrument movement at the very surface of the abdomen, may contribute to its favorable use. The Alexis wound retractor also acts as a wound protector to prevent tumor cell seeding or other port‐site contamination,^34^, ^36^, ^43^ and the glove can be disconnected from the wound retractor to exteriorize specimens. The disadvantages of the glove‐port technique include potential glove bulging or piercing by a needle or instruments, especially during long surgeries.^36^, ^40^ Some authors suggest the use of a double layer of gloves.^34^, ^35^, ^42^, ^43^

The main limitation of our study is the inclusion of historical data generated by other surgeons in different clinical settings. The senior surgeon and the assistant surgeon in our study were not familiar with the glove technique, but the senior surgeon had a broad experience of laparoscopic surgery. Thus, the feasibility of OHE with the glove‐port technique and nonarticulated instruments by novices remains unknown. In the future, a randomized prospective study including dogs surgically treated under controlled conditions with the glove technique or, for instance, with the SILS port would be interesting. Finally, the effect of resterilization on the Alexis wound retractor remains unknown and must be evaluated in the future.

In conclusion, our study provides evidence to support the feasibility of a single‐port LOHE with the glove‐port technique and nonarticulated instruments. The LigaSure device was used successfully to seal the suspensory and broad ligaments and cervix up to 18 mm in diameter. No conversion or major complications occurred. The glove‐port technique is a low‐cost alternative to other commercially available single‐port devices.

## CONFLICT OF INTEREST

The authors declare no conflicts of interest related to this report.
